# Injectable Crosslinked Genipin Hybrid Gelatin–PVA Hydrogels for Future Use as Bioinks in Expediting Cutaneous Healing Capacity: Physicochemical Characterisation and Cytotoxicity Evaluation

**DOI:** 10.3390/biomedicines10102651

**Published:** 2022-10-20

**Authors:** Syafira Masri, Manira Maarof, Nor Fatimah Mohd, Yosuke Hiraoka, Yasuhiko Tabata, Mh Busra Fauzi

**Affiliations:** 1 Centre for Tissue Engineering Centre and Regenerative Medicine, Faculty of Medicine, Universiti Kebangsaan Malaysia, Kuala Lumpur 56000, Malaysia; 2 Kumpulan Perubatan Johor Ampang Puteri Specialist Hospital, Kuala Lumpur 68000, Malaysia; 3 Biomaterial Group, R&D Center, Yao City 581-0000, Japan; 4 Laboratory of Biomaterials, Department of Regeneration Science and Engineering, Institute for Life and Medical Science (LiMe), Kyoto University, Kyoto 606-8500, Japan

**Keywords:** injectable hydrogel, 3D-bioprinting, bioinks, gelatin, PVA, skin tissue, wound healing

## Abstract

The irregular shape and depth of wounds could be the major hurdles in wound healing for the common three-dimensional foam, sheet, or film treatment design. The injectable hydrogel is a splendid alternate technique to enhance healing efficiency post-implantation via injectable or 3D-bioprinting technologies. The authentic combination of natural and synthetic polymers could potentially enhance the injectability and biocompatibility properties. Thus, the purpose of this study was to characterise a hybrid gelatin–PVA hydrogel crosslinked with genipin (GNP; natural crosslinker). In brief, gelatin (GE) and PVA were prepared in various concentrations (*w*/*v*): GE, GPVA3 (3% PVA), and GPVA5 (5% PVA), followed by a 0.1% (*w*/*v*) genipin (GNP) crosslink, to achieve polymerisation in three minutes. The physicochemical and biocompatibility properties were further evaluated. GPVA3_GNP and GPVA5_GNP with GNP demonstrated excellent physicochemical properties compared to GE_GNP and non-crosslinked hydrogels. GPVA5_GNP significantly displayed the optimum swelling ratio (621.1 ± 93.18%) and excellent hydrophilicity (38.51 ± 2.58°). In addition, GPVA5_GNP showed an optimum biodegradation rate (0.02 ± 0.005 mg/h) and the highest mechanical strength with the highest compression modulus (2.14 ± 0.06 MPa). In addition, the surface and cross-sectional view for scanning electron microscopy (SEM) displayed that all of the GPVA hydrogels have optimum average pore sizes (100–199 μm) with interconnected pores. There were no substantial changes in chemical analysis, including FTIR, XRD, and EDX, after PVA and GNP intervention. Furthermore, GPVA hydrogels influenced the cell biocompatibility, which successfully indicated >85% of cell viability. In conclusion, gelatin–PVA hydrogels crosslinked with GNP were proven to have excellent physicochemical, mechanical, and biocompatibility properties, as required for potential bioinks for chronic wound healing.

## 1. Introduction

The skin serves as the body’s primary protective barrier [1]. The skin helps regulate body temperature, maintains water and electrolyte levels, and detects sensation. As the largest organ in the body, the skin plays an important function in maintaining the body’s physiological haemostasis towards skin injury [2]. Skin injury refers to any damage or wound to the skin surface. Generally, wounds can be classified as acute wounds due to burns, trauma, radiation, and surgery. In contrast, chronic wounds usually occur due to illnesses such as diabetes, obesity, ulcers (pressure and diabetic foot ulcers), or impeded healing of the acute wound. Chronic wounds affect patients’ quality of life worldwide and are a significant cost increment in healthcare, especially wound care management. The rising prevalence and expenses have caused scrutiny. The worldwide treatment market is estimated to reach USD 4.8 billion in 2026, expanding at a compound annual growth rate (CAGR) of 6.4% from 2019 to 2026 [3].

Furthermore, over 50% of chronic wounds display signs and symptoms associated with localised bacterial biofilms underlying severe infections, which lead to tissue damage, delayed wound healing, and other major consequences [4]. Chronic wounds have been classified into categories based on their etiology, including pressure, diabetic, venous, and arterial, by the Wound Healing Society [5]. For example, pressure ulcers develop due to pressure or shear forces applied to the skin overlying bony prominences, resulting in ischemia/reperfusion and tissue damage. However, older adults tend to easily get pressure ulcers due to a lack of nutrients and chronic diseases that are caused by a multiple risk factor. Severe burns, venous ulcers, diabetic foot ulcers, and pressure ulcers are all associated with persistent tissue damage [6,7]. On another note, diabetes is a chronic condition caused by pancreatic dysfunction, which impairs normal insulin synthesis, resulting in high and variable blood glucose levels [4]. Diabetes mellitus (DM) is a metabolic disorder that affects almost every country. In 2019, it was predicted that 463 million individuals worldwide were suffering from diabetes and the number is expected to increase in the following years [8]. Ineffective therapeutic intervention in diabetic patients can lead to long-term complications such as neuropathy, retinopathy, atherosclerosis, and nephropathy, leading to other comorbidities such as delayed wound healing. Therefore, the autologous full-thickness or split-thickness (meshed or non-meshed) skin transplant provides the best covering especially for burn wounds [9]. However, the main restriction for skin graft treatment is insufficient of donor site tissue for the patients. Moreover, the irregular shape and various depths could be major challenges for currently available treatments. 

Therefore, tissue engineering (TE) aims to create functional alternatives for injured tissue by integrating biology and engineering concepts [10]. The ultimate goal for tissue injury is to promote tissue regeneration, which helps to restore the structure and function of the native tissue as faithfully as is feasible [11]. In recent decades, TE introduced a new concept of a fabrication technique using injectable hydrogels to deliver cells to specific lesion areas. It is often assumed that cells encapsulated in hydrogels perceive their biomechanical surroundings via focal adhesion [12]. In addition, hydrogels are polymers that can hold a large volume of water [13]. The porous structure of hydrogels can aid in the absorption of wound exudate, thus reducing the risk of infection and promoting a conducive environment for wound healing [14]. 

Nowadays, conventional fabrication techniques have given rise to many of the 3D-bioprinting methods. In general, 3D-bioprinting involves using several printing techniques to dispense the bioinks. Briefly, the extrusion-based bioprinters deposit bioinks to form a 3D biomatrix; droplet-based bioprinting offers precise depositions of bioinks droplets; and laser-assisted bioprinting uses laser technology to transfer bioinks to a substrate in a 3D spatial arrangement [15]. The “bioinks”, which is mostly composed of biomaterials, live cells, and/or bioactive chemicals, is applied in a predesigned layer-by-layer fashion to a free-moving platform to create geometrically well-defined 3D complex structures [16]. The use of “bioinks” as a cellular treatment helps in better cell differentiation for organ construction and regeneration [17]. 

To date, smart biomaterials, including natural and synthetic polymers, have been explored extensively in the biomedical area, where changes in the body may be used to influence their responsive behaviour. In addition, they often display key behaviour such as biodegradability, biocompatibility, and highly mimic the native extracellular environment [18]. Recently, thermoresponsive polymers have recently gained more attention due to their distinctive ability to switch from the liquid to solid phase (sol-gel) at a temperature change and have shown the potential of bioprinted thermoresponsive constructs [19]. Thermo-responsive polymers such as agarose, gellan gum, collagen, gelatin, Poly(N-isopropylacrylamide), polyvinyl-alcohol (PVA), and poloxamer have temperature-dependent viscosity, which can be adjusted through a nozzle temperature controller [20,21].

Gelatin is traditionally derived from fish skin collagen (mainly type I), bovine, and porcine. Collagen is the most naturally occurring protein in humans and animals as a source of the protein-matrix of gelatin [21]. Acidic or basic hydrolysis of collagen results in type A or type B gelatin [22]. An isoelectric point of type A gelatin at pH 6 to 9 is most typically used for the less covalently crosslinked collagen present in pig skin, whereas type B gelatin is derived from bovine sources [23]. The bioactive sequences of gelatin in particular are generated from collagen (e.g., MMP-sensitive degradation sites and RGD peptides) [24]. The advantages of using gelatin as a biomaterial are non-toxicity, non-carcinogenicity, biocompatibility, and biodegradability [25]. Apart from its advantages as a natural protein-based hydrogel, gelatin is also cheap, as it is often produced from manufacturing by-products such as skins and bones [13]. To date, most of the studies have developed potential bioinks using gelatin with other types of polymers. In the present study, an alginate–gelatin-based hydrogel was employed as the bioinks with a combination of fibrinogen (FIB) and diethyl aminoethyl cellulose (DCEL) for prospective 3D-bioprinting skin applications [26]. The study hypothesised that gelatin and fibrinogen both contribute to biological activities that improve biocompatibility. In addition, a blending of gelatin–alginate has also received high attention as a potential bioinks for wound healing [27]. The study revealed that the gelatin–alginate bioinks promoted MSCs proliferation, migration, and differentiation via the 3D-bioprinting process. Moreover, the biocompatibility of these hydrogels in terms of gelatin content was tested in vitro in mouse fibroblast cells. The fibroblasts adherence in the hydrogels increased as the gelatin concentration increased [28]. 

A composite scaffold composed of both natural and synthetic biomaterials may enable the generation of skin substitutes for different wound sizes, degrees of burn, patient ages, and availabilities of fabrication techniques that meet all clinical requirements. Natural biopolymers have the ability to enhance cell responsiveness, while synthetic polymers offer greater control over chemical composition and mechanical properties [29]. It is hypothesised that using one polymer only sometimes cannot satisfy all of the criteria for a skin replacement. Therefore, polyvinyl-alcohol (PVA) is a widely used and commercialised synthetic polymer in the tissue engineering field. PVA is known as a water-soluble and biocompatible synthetic polymer utilised in wound dressing and medication delivery systems [30]. The combination of gelatin and PVA may aid in the improvement of mechanical properties and muco-adhesiveness [30]. The mechanical properties of PVA rely on thermal transition, in which a PVA solution crystallises via non-covalent intermolecular interactions to develop a crosslinked polymer network structure [31]. The hydrogen bond formation between gelatin–PVA was postulated, resulting in high resilience of the hydrogel at the site of action due to the supramolecular network. A study finding demonstrated that PVA has an important function in increasing water absorption and mechanical strength, as well as controlling the rate of biodegradation [32]. 

Genipin is known as a natural crosslinker that is frequently employed for drug ad-ministration due to its great properties, including being a biocompatible, biodegradable, non-toxic and stable crosslinker [33,34]. Cytotoxicity testing on genipin against fibroblasts revealed that genipin was 10,000-fold less harmful than glutaraldehyde, and fibroblasts proliferated 5000-fold more than when glutaraldehyde was used [35]. Genipin, a natural compound obtained from Gardenia plants, was demonstrated to improve the physicochemical properties of the gelatin scaffold [36]. Previous research has demonstrated that the genipin crosslinker promotes fibroblasts adhesion and proliferation, making it a promising candidate for tissue engineering applications. A previous study demonstrated that the fabrication of gelatin crosslinked with genipin was effectively biocompatible with excellent cell viability [37]. The exploration of genipin on gelatin scaffold is expanding rapidly with many current active studies and projected to rise shortly with the effective clinical deployment of gelatin-based products.

In this work, an injectable hybrid gelatin–PVA hydrogel crosslinked with genipin was fabricated as a potential biomatrix for chronic wound treatment. The properties of GPVA hydrogels were compared between non-crosslinked and crosslinked groups. The physicochemical, mechanical, and cytotoxicity were evaluated. This innovation was aimed to enhance the mechanical properties of the hydrogels as potential bioinks for future 3D-bioprinting applications. This cellular treatment possesses a benefit to promote the proliferation of the cells in the chronic wound area. As a result, the current study hypothesised that the bilayer scaffold might be a promising candidate for future bioinks in wound care management.

## 2. Study Design

The study design was approved by the Universiti Kebangsaan Malaysia Research Ethics Committee (Code no. FF-2021-376 and JEP-2021-605).

### 2.1. Hydrogel Preparation

Polyvinyl Alcohol (PVA) powder (partially hydrolysed ≥85% and Mw 70,000 g/mol) was obtained from (MERCK KGaA, Darmstadt, Germany) was dissolved in distilled water (dH_2_O) at 60 °C, and stirred for 1 h until a homogeneous solution was obtained at 3% and 5% (*w*/*v*) concentration. Gelatin (GE) powder (Nitta-Gelatin Ltd., Yao City, Osaka, Japan) at 6% (*w*/*v*) concentration was added to the solution and continuously stirred for 30 min at 40 °C to achieve a homogenous solution. Genipin (GNP) solution at 0.1% (*w*/*v*) concentration was made by dissolving GNP powder (FUJIFILM Wako Pure Chemical Corporation, Chuo-Ku, Osaka, Japan) with 70% ethanol (EtOH; MERCK, Darmstadt, Germany). The GNP solution was then added into the solution to obtain final formulation of GE_GNP (0.1% GNP), GPVA3_GNP (3% PVA_0.1% GNP), and GPVA5_GNP (5% PVA_0.1% GNP), whereas the non-crosslinked hydrogels were represented as GE_NC, GPVA3_NC (3% PVA), and GPVA5_NC (5% PVA). The polymerisation time of the hydrogels was recorded at ±23 °C using the inverted tube test method. For the 3D-bioprinting fabrication technique, the bioinks was loaded into the syringe and printed at 23 ± 2 °C using an extrusion 3D-bioprinter (Biogens XI).

### 2.2. Evaluation of Gross Appearance

The gross appearance, including the top and cross-sectional view of the fabricated hydrogels via injectable technique for both non-crosslinked and crosslinked hydrogels, was taken immediately after polymerisation using a digital camera (Nikon, Tokyo, Japan). In addition, the gross appearance of the potential bioinks was evaluated and fabricated using a 3D-bioprinter (BiogensX1).

### 2.3. Swelling Ratio

The swelling behaviour of the hydrogel was observed, adapted from a previous study [37]. The swelling ratio was calculated to determine the ability of hydrogels to absorb wound exudates. In brief, the freeze-dried hydrogels were weighed (Wi) before being submerged in phosphate buffer saline (PBS, pH = 7.4) at room temperature for 6 h. Excess buffer was removed using filter paper, and the final weight (Wf) of the hydrogel was weighed. The percentage of swelling ratio was calculated using the following formula:

$$\mathrm{Swelling}\;\mathrm{Ratio}\;\left(\%\right)=\frac{\left(W2-W1\right)}{W1}\times 100$$

where W2 is the weight of the hydrogels after immersion and W1is the weight of the hydrogels before immersion.

### 2.4. Enzymatic Degradation

The enzymatic biodegradation analysis evaluated the hydrogels’ biodegradability after exposure to the enzymatic reaction. The analysis was carried out by weighing the hydrogels before immersing them in 0.0006% (*w*/*v*) collagenase type-I (Worthington, Lakewood, NJ, USA) in a 24-well plate and incubating them at 37 °C for 6 h. We removed the enzyme and rinsed the hydrogels with distilled water to eliminate residual salts in the porous structure before weighing them to determine the final weight (W2) of the hydrogels. The percentage of weight loss was calculated using the following equation:

$$\mathrm{Weight}\;\mathrm{Loss}\;\left(\%\right)=\frac{\left(W2-W1\right)}{W1}\times 100$$

where W2 is the final weight and W1 is the initial weight.

### 2.5. Contact Angle

The contact angle was utilised to determine the hydrophilicity of the polymerised hydrogels. The ImageJ Software (National Institute of Health, V1.5, Bethesda, MA, Maryland USA) was used to analyse the contact angle of the hydrogels. A drop of 10 μL of distilled water was dropped onto the surface of the hydrogels, and the images were captured using a digital camera.

### 2.6. Water Vapor Transmission Rate

The hydrogels were subjected to a water vapor transmission rates (WVTR) test to determine their ability to transmit water and allow gases’ exchange through the hydrogel to aid in wound healing, in accordance with the American Society for Testing and Materials (ASTM) standard [38,39]. Briefly, the hydrogel was placed onto a cylindrical cup with 10 mL of distilled water. The samples were then put in a controlled atmosphere of 5% CO_2_ at 37 °C in an incubator. The following formula was used to record and evaluate the results:

$$\mathrm{WVTR}=\frac{\left(\mathrm{Wi}-\mathrm{Wf}\right)}{\left(A\;\times \;\mathrm{Time}\right)}$$

where Wi is the initial weight, Wf is the final weight, and A is the surface area of the cylinder bottle.

### 2.7. Mechanical Testing

The mechanical testing was adapted from Salleh et al., 2022, with some modification using a simple compression test [38,39]. The sample compression was measured by placing the polymerised hydrogel at room temperature. The hydrogels used were approximately 2 cm in diameter with 3 mm in height. The compression modulus (E) was measured using the formula below [40,41]:

$$E=\frac{\sigma }{\varepsilon }$$

σ= compressive force per unit area (stress)

ε= changes in volume per unit volume (strain)

### 2.8. Viscosity

To assess the viscosity of the hydrogels, the experiment was performed in triplicate using a viscometer (Brookfield DVE Digital Viscometer) at a temperature of 23 °C using a cylindrical spindle (LV1; 18.84 mm diameter and 65.10 mm length). The hydrogels were prepared at 37 °C and allowed to cool down to 23 ± 2 °C before being tested with the viscometer. The viscosity reading was then recorded.

### 2.9. Resilience

The scaffolds’ resilience, their ability to retain their original shape after being subjected to pressure, was adapted from Salleh et al., 2022 [39]. A total of 300 g of metal weight was imposed on the bio-composite scaffolds for 2 min. The bioscaffolds were then immersed in distilled water for 2 min. ImageJ software (NIH, Bethesda, Maryland, USA) was used to record and evaluate the area of thickness before compression, after compression, and after rehydration. The resilience (R) was calculated using the formula:

$$\mathrm{Resilience}\;\left(\%\right)=\frac{\mathrm{Ai}-\mathrm{Ac}}{\mathrm{Af}}\times 100$$

where Ai is the area of thickness before compression, Af is the area of thickness after rehydration, and Ac is the area of thickness after compression.

### 2.10. Degree of Crosslinking

The degree of crosslinking of hydrogels was measured using the Ninhydrin Assay (Sigma Aldrich, Saint Louis, MO, USA). The free amino groups of crosslinked hydrogels that interacted with GNP were compared to non-crosslinked hydrogels. A serial dilution technique was used to generate the glycine standard curve (0.006, 0.0125, 0.025, 0.05, and 0.1 mg/ mL). Firstly, the hydrogels were lyophilised for 24 h. The hydrogels were initially weighed at 10 mg for each scaffold. The samples were then boiled at 100 °C for 2 min according to the package recommendations. The free amino groups for both non-crosslinked and crosslinked hydrogels were measured by using a spectrophotometer (BioTek, PowerWave XS, Highland Park, Winooski, Vermont, IL, USA) with optical absorbance at 570 nm (Abs 570). The degree of crosslinking was then calculated using the following formula:

$$\mathrm{Degree}\;\mathrm{of}\;\mathrm{Crosslinking}=\frac{\mathrm{Anc}-\mathrm{Ac}}{\mathrm{Anc}}\times 100$$

where Anc is the absorbance of the non-crosslinked hydrogel and Ac is the absorbance of the crosslinked hydrogel.

### 2.11. Porosity Measurement

The porosity percentage was calculated using freeze-dried hydrogels from two different methodologies, as described in further detail below.

#### 2.11.1. Scanning Electron Microscopy (SEM)

The microstructure of hydrogels was observed using field-emission scanning electron microscopy (FESEM; Supra 55VP model, Jena, Germany). ImageJ software (V1.5, Bethesda, Maryland, USA) was then used to calculate the average pore diameters. Prior to analysis, the lyophilised hydrogels were coated with an ultra-thin coating of gold using ion sputtering. 

#### 2.11.2. Liquid Displacement Method

Ethanol is a non-polar liquid that does not interact with polymeric fibres; hence, it simply penetrates the scaffold and occupies all of the holes in the sample, yielding the total volume of pores. The freeze-dried hydrogels were submerged in a liquid that did not disintegrate or swell the scaffolds for this method. The weight of the scaffolds before and after immersion in ethanol was measured. The following equation was used to calculate the percentage of porosity:

$$\mathrm{Porosity}\left(\%\right)=\frac{\left(\mathrm{Wf}-\mathrm{Wi}\right)}{\rho V}\times 100$$

where Wf, Wi, and V indicate the weight of the final scaffold, the weight of the initial scaffold, ρ is ethanol density (0.789 g/m^3^), and the volume of the scaffold, respectively.

### 2.12. Surface Roughness

An Atomic Force Microscope (AFM) was used to characterise the surface roughness of the lyophilised hydrogel using an AFM analyser (Park Systems, NX-10, Suwon, Korea). The AFM images were analysed with the XE Image Processing Program, and the roughness of the scaffold surface was calculated. Surface roughness testing was carried out on a 5 × 5 mm sample using non-contact mode scanning at 0.2 Hz (scan size 5 and 2 nm) and pixel 256 × 256.

### 2.13. Sample Characterisation

The hydrogels’ functional groups were determined using a Fourier Transform Infrared (FTIR) spectrometer (PE, Waltham, Maryland, USA) with a wavelength range of 4000 cm^−1^ to 500 cm^−1^. The absorbance peaks were analysed in order to determine the chemical structure and changes that occurred after crosslinking. Furthermore, an energy dispersive X-ray (EDX) analysis was carried out to assess the presence of the element’s composition in the hydrogels. A Phenom Pro X SEM EDX microscope (Phenom, Eindhoven, the Netherlands) was used to perform this analysis. The commercial gelatin, GNP, and PVA powder were used as a control. The crystallinity of the hydrogels was evaluated using an X-ray diffractometer (Bruker, D8 Advance, Coventry, UK) with a diffraction angle of 2θ in the temperature range of 0 °C to 80 °C. The diffractogram was then further evaluated by using the integrated software (Diffrac. Suite EVA, V4.0, Bruker, Coventry, UK).

### 2.14. Thermostability Analysis

Thermogravimetric analysis (TGA) was performed using a TGA (Shimadzu, model TGA-50). The dynamic tests were performed in a nitrogen-contained environment within a range of 25–200 °C to estimate the thermal stability of the hydrogels when they were heated up at a constant rate (10 °C/min). The sample weight loss as a function of temperature was recorded continuously. The result obtained was analysed using ta60w software.

### 2.15. Skin Cell Isolation and Culture

**Figure 1:**
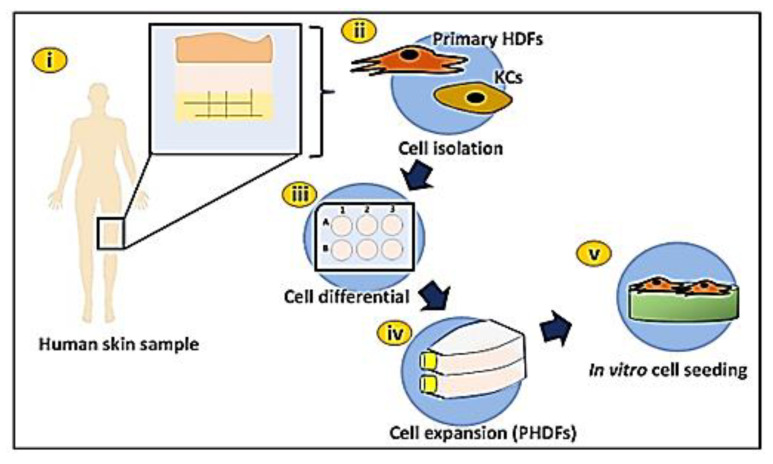
Experimental design for human skin cell isolation: (**i**) collection of human skin sample, (**ii**) cell isolation: primary HDFs and KCs, (**iii**) cell differential prior primary HDFs expansion, (**iv**) primary HDFs expansion in T75 cell culture flasks, (v) in vitro cell seeding (primary HDFs) on hydrogel.

To construct a bioengineered scaffold for chronic wound patients, primary human dermal fibroblasts (PHDFs) were isolated from human skin samples collected as redundant tissue after surgery from three consenting patients. Skin samples from different patients were further processed separately by cutting them into small (1–2 cm) pieces and cleaning them using sterile Dulbecco’s Phosphate Buffer Saline (DPBS). They were then digested with 0.6% collagenase Type I (Worthington-Biochemical Corporation, 730 Vassar Ave Lakewood) for 4–6 h at 37 °C in the shaker incubator, followed by the trypsinization process using trypsin-EDTA (Gibco/BRL, Carlsbad, CA, USA) for 10 min. The cell suspension was then centrifuged before being resuspended in a co-culture medium comprising Epilife (Gibco/BRL, Carlsbad, CA, USA) and F12:DMEM (Gibco/BRL, Carlsbad, CA, USA) in the same ratio (1:1), which was supplemented with 10% Fetal Bovine Serum (FBS) (Biowest, Bradenton, USA). The cells were then seeded at 1 × 10^4^ cells/cm^2^ in a 6-well polystyrene culture plate and incubated at 37 °C in 5% CO_2_. The medium was changed every 2–3 days prior to differential trypsinization after the cells achieved 70–80% confluency. The HDFs was expanded in a 75 cm^2^ culture flask containing F12:DMEM with 10% FBS. Figure 1 shows the experimental design for primary human dermal fibroblasts isolation.

An in vitro biocompatibility test of cultivated primary HDFs was evaluated using the LIVE/DEAD cytotoxicity for mammalian cells (Thermo Fisher Scientific, Waltham, MA, USA). Sterile gelatin–PVA hydrogels were fabricated in a 48-well polystyrene culture plate to form crosslinked hydrogels. Then, 35 × 10^3^ HDF passage three were seeded on top of the hydrogel immediately after polymerisation, before incubation for 24 h. After 30 min of treatment with 250 μL of a mixture of 2 mM acetomethoxy calcein derivate (calcein-AM) and 4 mM ethidium homodimer-1 (EthD-1) at 37 °C, the cell cytotoxicity of the HDFs was measured using a fluorescence microscope (Nikon A1R-A1, Japan) at 100× magnification.

### 2.16. Cell Morphology

Scanning electron microscopy (FESEM; Supra 55VP model, Jena, Germany) was used to observe the HDFs morphology after interaction with the hydrogels. The hydrogels were fixed with 4% paraformaldehyde (PFA) overnight after being seeded with HDFs (1 × 10^4^/cm^3^). The dehydration of the hydrogels was adapted from Busra et al., 2017, using immersion in a series of ethanol solutions (30%, 50%, 70%, and 100%; 10 min each) [42]. The hydrogels were lyophilised using a freeze-dryer overnight before being sputter-coated with nanogold for SEM examination.

### 2.17. Statistical Analysis

All the reported data were analysed using Graph Pad Prism (V9.0, GraphPad Software Inc., San Diego, CA, USA). One-way and two-way ANOVA was used for the multiple groups analysis. The mean ± standard deviation of all data was calculated. The *p* value of 0.05 was used to determine statistical significance. All quantitative data values were obtained from three (*n* = 3) replicate trials.

## 3. Results

### 3.1. Gross Appearance and Polymerisation Time

**Figure 2:**
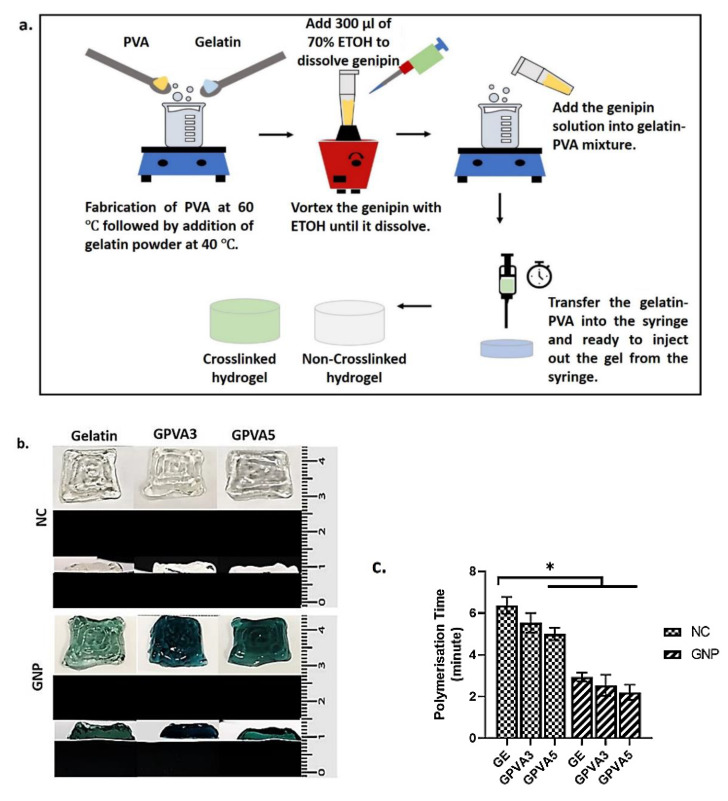
(**a**) Schematic of the hydrogel fabrication steps, (**b**) gross appearance (top view and cross-sectional) of hydrogel, and (**c**) polymerisation time for non-crosslinked and crosslinked hydrogels, where * indicates *p* < 0.05.

Gelatin–PVA hydrogels were fabricated and chemically crosslinked using genipin (GNP). Figure 2a,b,d shows the fabrication procedures, gross appearance including top view and cross-sectional view, and polymerisation time of GE NC, GPVA3 NC, GPVA5 NC, GE GNP, GPVA3 GNP, and GPVA5 GNP, respectively. Non-crosslinked hydrogels were colourless, whereas crosslinked hydrogels were green. The polymerisation time of hydrogels was observed within three-minute periods at 22–24 °C. In addition, Figure 2c demonstrates the gross appearance of the printed hydrogels that were successfully fabricated using a 3D-bioprinter. The major prerequisite for selecting bioinks for future 3D-bioprinting hydrogels is an appropriate formulation to improve printability and maintain cell viability for post-bioprinting.

### 3.2. Physicochemical Analysis

**Figure 3:**
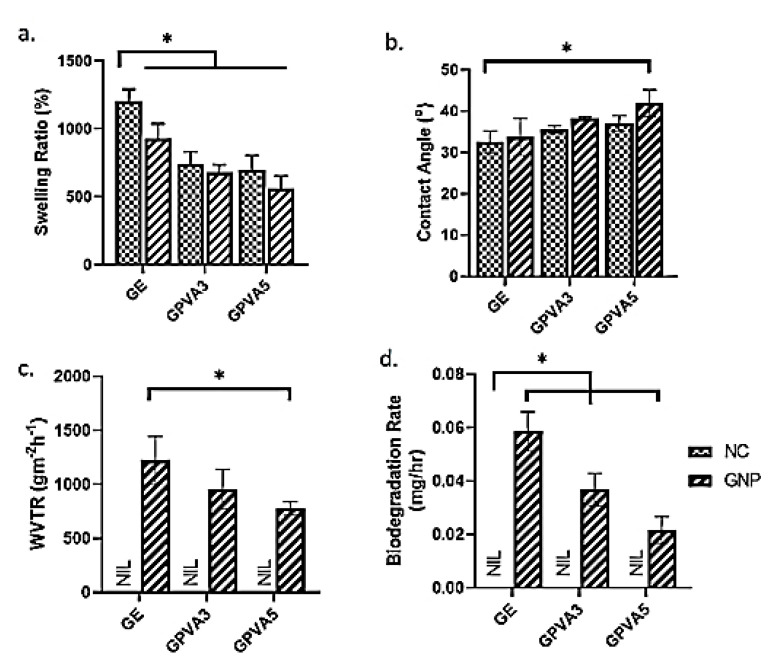
Physicochemical analysis of GE, GPVA3, and GPVA5 (**a**) % of swelling ratio (°), (**b**) contact angle, (**c**) water vapor transmission rate (WVTR) (gm^−2^h^−1^), and (**d**) biodegradation rate. * indicates *p* < 0.05.

The physicochemical properties of gelatin–PVA hydrogels were evaluated via contact angle, water vapor transmission rate (WVTR), biodegradation rate, swelling ratio, and pore size. The quantitative measurements were scrutinised for both crosslinked and non-crosslinked hydrogels in Figure 3. Hydrogel wound dressings should have a high-water absorption capacity to absorb wound exudates. The GPVA hydrogels were revealed to have optimum percentages of swelling properties after the addition of PVA and GNP, at >500%. Based on the result, the crosslinked hydrogels can hold water in the range of 500% to 800% in the fluid. GE_NC showed the highest water absorption ability with 1232.04 ± 42.10 followed by GE_GNP with 965.78 ± 155.38%. Hydrogels that incorporated with PVA demonstrated significantly lower swelling ratio compared to GE_NC. GPVA3_GNP and GPVA5_GNP exhibited swelling ratios of 688.59 ± 56.86% and 621.05 ± 93.18%, respectively.

The contact angle in Figure 3b demonstrates that both non-crosslinked and crosslinked GPVA hydrogels exhibited a lower contact angle (°), which is less than 90°. GE_NC exhibited the lowest contact angle, which is 32.58 ± 2.67°, significantly lower than the crosslinked hydrogels. Meanwhile, the crosslinked hydrogels GPVA3_GNP and GPVA5_GNP have higher contact angles (38.18 ± 0.40°, and 38.51 ± 2.58°) compared to GE_GNP (33.78 ± 4.49°). In comparison, there are no significant differences between all crosslinked hydrogels.

WVTR analysis, demonstrated in Figure 3c, indicated that crosslinked gelatin–PVA hydrogels have the capability to allow the water vapor to penetrate throughout the crosslinked hydrogels within the acceptable range, which is below 1500 gm^−2^h^−^^1^. The highest WVTR was exhibited by GEL_GNP, resulting in 1061.48 ± 318.48 gm^−2^h^−1^. Increasing the amount of PVA in the hydrogels led to decrease in the WVTR. Therefore, GPVA3_GNP and GPVA5_GNP demonstrated slightly lower WVTRs (955.47 ± 183.83 gm^−2^h^−1^ and 778.51 ± 61.28 gm^−2^h^−1^), respectively. The quantitative measurement of the biodegradation rate of the gelatin–PVA hydrogels in Figure 3d demonstrated that the GPVA5_GNP hydrogel exhibited a significantly lower biodegradation rate (0.022 ± 0.005 mg/h) compared to other crosslinked hydrogels, GE_GNP and GPVA3_GNP (0.058 ± 0.007 mg/h and 0.037 ± 0.006 mg/h). However, non-crosslinked hydrogels were fully degraded within an hour after being exposed to the enzymatic reaction. 

### 3.3. Mechanical Strength of the Fabricated Hybrid Hydrogel

**Figure 4:**
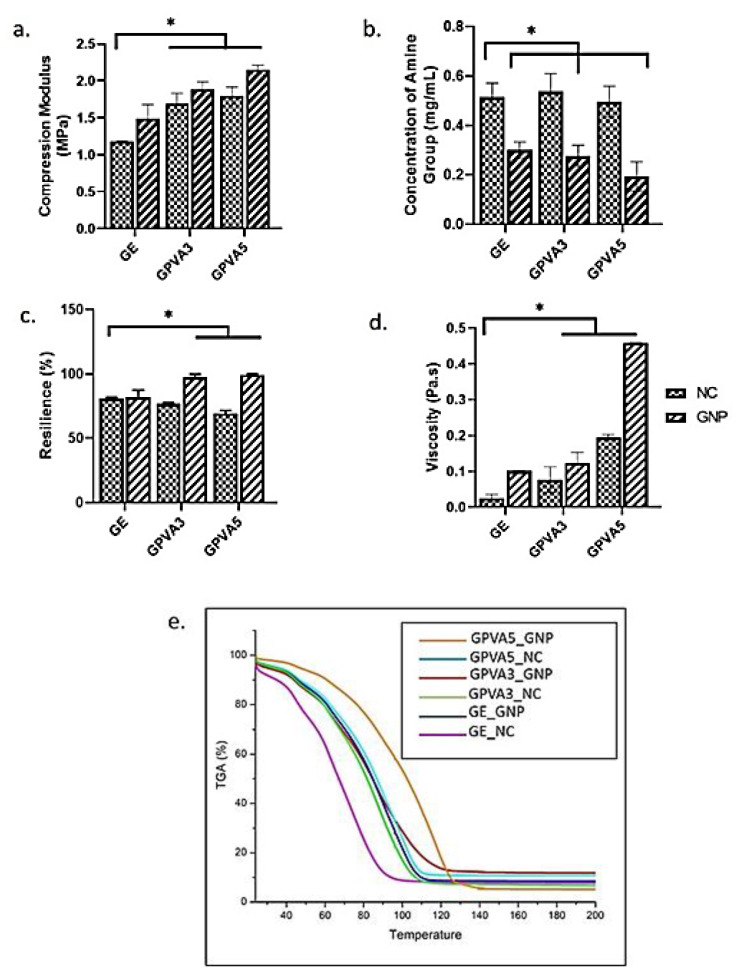
Mechanical properties analysis of gelatin, GPVA3, and GPVA5: (**a**) compression modulus, (**b**) concentration of amine groups, (**c**) percentage of resilience, (**d**) viscosity, and (**e**) TGA analysis, where * indicates *p* <0.05.

Mechanical strength is essential to withstanding pressure during biomatrix implantation at the wound site. Figure 4a shows the compression analysis used to evaluate the mechanical strength of the hydrogels. GPVA5_GNP demonstrated a significantly higher compression modulus compared to the non-crosslinked hydrogels. In addition, the crosslinked GPVA5_GNP hydrogel was revealed to have the highest percentage of compression (2.14 ± 0.06 MPa). The crosslinked hybrid hydrogel presented a low crosslinking degree with a low presence of free amine groups compared to the non-crosslinked group, as shown in Figure 4b. The hydrogel’s resilience was evaluated to test the ability of the hydrogel to return to its original shape after being subjected to compression or pressure. The resilience of the crosslinked hydrogels that were incorporated with PVA demonstrated higher resilience compared to the non-crosslinked hydrogels, and the values of GE_NC, GPVA3_NC, GPVA5_NC, GE_GNP, GPVA3_GNP, and GPVA5_GNP are 76.03 ± 4.86%, 77.03 ± 6.80%, 74.67 ± 7.66%, 96.66 ± 2.01%, and 91.33 ± 0.58%, respectively, as shown in Figure 4c. 

Prior to 3D-bioprinting application, the hydrogel with optimum viscosity was highly suggested to ensure printability quality throughout the bioprinter nozzle. The crosslinked hydrogels have higher viscosity compared to the non-crosslinked group, and the values of GE_NC, GPVA3_NC, GPVA5_NC, GE_GNP, GPVA3_GNP, and GPVA5_GNP are 0.026 ± 0.009 Pa.s, 0.07 ± 0.03 Pa.s, 0.195 ± 0.008 Pa.s, 0.11 ± 0.001 Pa.s, 0.124 ± 0.002 Pa.s, and 0.458 ± 0.001 Pa.s, respectively, as shown in Figure 4d. The TGA curves of the gelatin–PVA hydrogels are shown in Figure 4e and values for the onset temperatures were taken. All samples exhibited a major mass loss stage in the temperature range of 25–200 °C.

### 3.4. Chemical Characterisation

**Figure 5:**
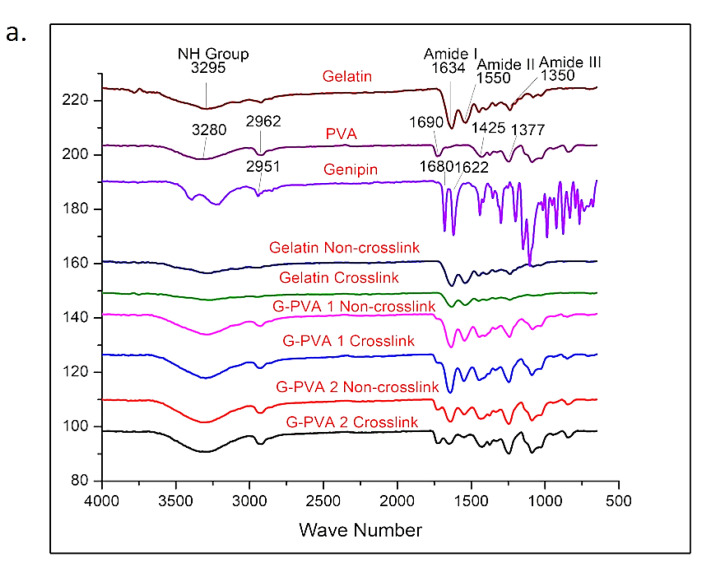

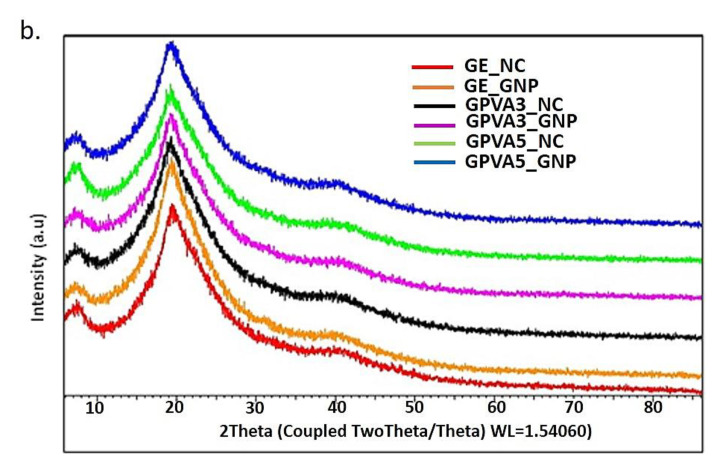
Chemical characterisation of hydrogel. (**a**) FTIR spectra of pure gelatin, PVA, genipin, and fabricated hydrogels, and (**b**) crystallinity of hydrogels via X-ray diffraction analysis (XRD).

The FTIR spectra in Figure 5a showed the presence of absorption peaks in gelatin at 3295 cm^−1^, which represent the presence of C-H stretches and N-H aliphatic. Furthermore, the occurrence of peaks at 1550 cm^−1^ and 1634 cm^−1^ was attributed to N-H bending (amide II) and amine C=O stretching (amide). Furthermore, the broader peaks of PVA at 3280, 2962, 1690, 1425, and 1377 cm^−1^ represent O-H stretching vibration of the hydroxy group, -CH2, asymmetric stretching vibration, C=O carbonyl stretch, C-H bending vibration of CH_2_, C-H deformation vibration, C-O stretching of acetyl groups, and C-C stretching vibration, respectively. In addition, GNP demonstrated the presence of an absorption peak at 1680 cm^−1^, which was assigned to carboxyl group (C-O) stretching vibration, and another absorption peak at 1622 cm^−1^, which was attributed to C-C vibration of the olefin ring in genipin. The peak that appeared in the genipin around 1800–3000 cm^−1^ was attributed to C-H stretching vibration. The double peak at 3000–3600 cm^−1^ in the genipin spectrum was most probably due to an overlapping of aromatic C-H and O-H vibration bands. 

The XRD profiles of gelatin–PVA hydrogels are represented in Figure 5b. The XRD test was frequently used to identify the crystalline phase of the scaffolds based on the diffraction peak orientation, peak pattern, and the measurement of the mean crystalline sizes of the gelatin, PVA, and genipin within the hydrogel network. As shown in Figure 5b, a broad peak at 20° is related to the polymer network. The XRD result demonstrated that the presence of gelatin, PVA, and genipin inhibited the crystallisation of crosslinked and non-crosslinked gelatin–PVA hydrogels. Table 1 shows the percentage of crystallinity and the amorphous level of hydrogels.

The elemental compositions of the hydrogels were determined by energy dispersive X-ray (EDX) spectroscopy, as shown in Table 2. The carbon (C) presence was attributed to gelatin and PVA polymers. The signals for EDX analysis showed that the addition of PVA into the gelatin hydrogels influenced the increase in the percentage of C components in the scaffolds. The crosslinked hydrogels resulted in a minor drop in carbon and an increase in oxygen components; nevertheless, no substantial change was observed.

### 3.5. 3D-Microporous Structure Hydrogel

**Figure 6:**
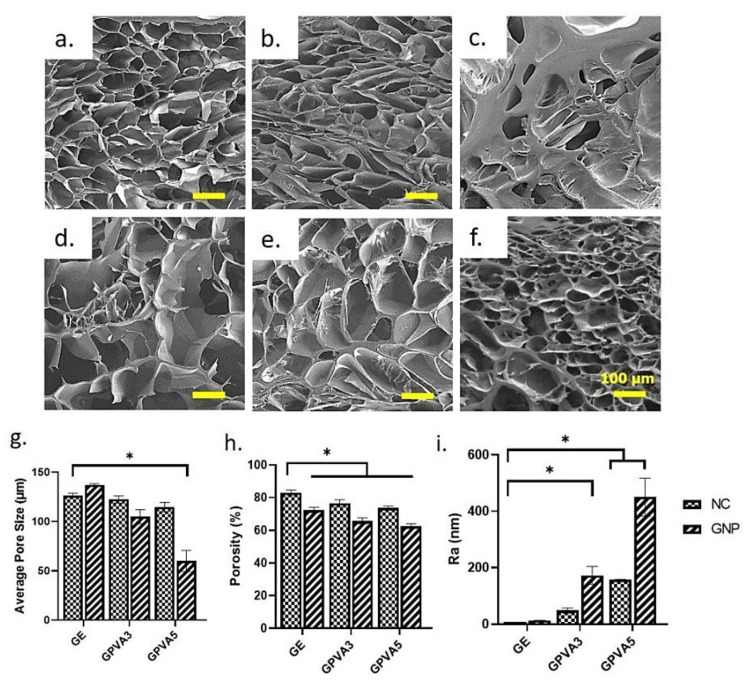

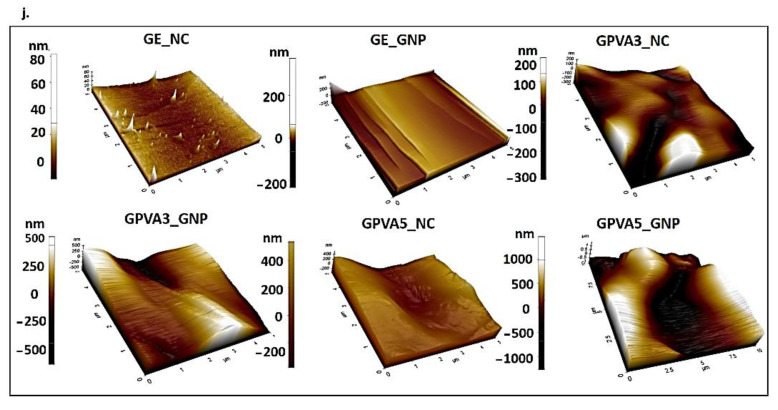
SEM images showing the cross-sectional microporous structure of the hydrogels: (**a**) GE_NC, (**b**) GPVA3_NC, (**c**) GPVA5_NC, (**d**) GE_GNP, (**e**) GPVA3_GNP, and (**f**) GPVA5_GNP under 100× magnification; (**g**) average pore size (μm); (**h**) porosity percentage; (**i**,**j**) AFM analysis for surface roughness. * Indicates significant difference.

The SEM micrographs of gelatin–PVA hydrogels, non-crosslinked and crosslinked, revealed the heterogenic porous structure via the cross-sectional SEM images in Figure 6a–f. The SEM images also demonstrated a more compact microstructure for non-crosslinked hydrogels, GE_NC, GPVA3_NC, and GPVA5_NC, compared to crosslinked hydrogels. All the images were analysed using ImageJ software. However, the crosslinked hydrogels have an acceptable porous and homogenous pore-like structure with high interconnectivity. The freeze-drying procedure resulted in mean pore sizes for GE_NC, GPVA3_NC, and GPVA5_NC hydrogels of 98.0 ± 44.8 μm, 112.6 ± 40.3 μm, and 111.82 ± 38.6 μm, respectively, whereas the GE_GNP, GPVA3_GNP, and GPVA5_GNP hydrogels demonstrated mean pore sizes of 134.7 ± 43.4 μm, 109.4 ± 38.5 μm, and 108.5 ± 24.5 μm, respectively, as shown in Figure 6g. The average pore sizes of the hydrogels decreased with increases in PVA concentration. It seems that a high concentration of PVA and the addition of a crosslinker in the polymer solution prevents the hydrogel from obtaining a uniform pore-like structure. However, the microstructure of the hydrogels was similar to that of native human skin tissue. The percentages of porosity of the hydrogels are described in Figure 6h. 

The surface topology of the hydrogel samples and their mean surface are given in Figure 6j. The surface roughness of hydrogels will influence the cell behaviour and cell adhesion activity. Gelatin–PVA hydrogels inherently have a rough topology. The Ra value in Figure 6i indicated that GE_NC (6.62 ± 0.32 nm) significantly has the highest Ra (nm) value with GPVA3_GNP, GPVA5_NC, and GPVA5_GNP having values of 172.53 ± 31.50 nm, 158.30 ± 0.52 nm, and 450.22 ± 67.68 nm, respectively. Moreover, GE_CL and GPVA3_NC showed the lowest Ra (nm) values at 13.37 ± 0.47 nm and 48.30 ± 9.05 nm, respectively. An increasing amount of PVA in hydrogels increases the mean of surface roughness. 

### 3.6. Cytotoxicity Assessment

**Figure 7:**
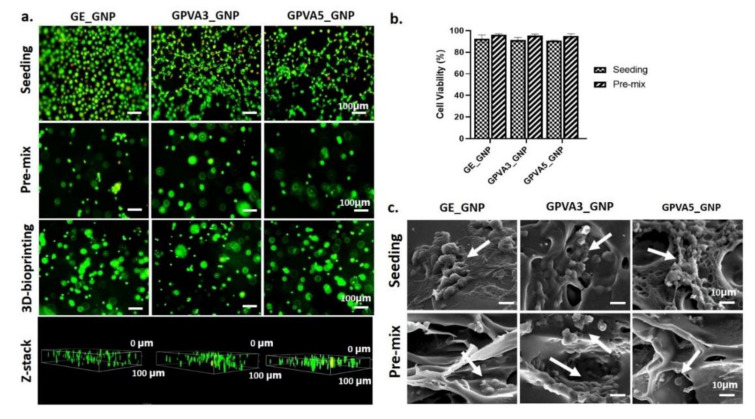
HDF cytotoxicity and interaction with hydrogels. (**a**,**b**) Live and dead assay and cytotoxicity evaluation of HDFs with hydrogel through seeding, pre-mix, and 3D-bioprinting techniques under 100× magnification. (**c**) Cell morphology on the surface of hydrogels, and after pre-mixing in the hydrogels, under 10× magnification.

Live and dead assay of HDFs cultured on the crosslinked hydrogels via cell seeding and pre-mixed approaches demonstrated no toxic effect after 24 h, as shown in Figure 7. Nevertheless, the morphology of the HDFs seeded on top of the GE_GNP hydrogel was a rounded shape, whereas the HDFs appeared as interconnected between cells on the G-PVA3_GNP and G-PVA5_GNP hydrogel surfaces. The green colour represents living cells, whereas the red colour represents dead cells.

Moreover, the morphology of the pre-mixed GE_GNP, GPVA3_GNP, and GPVA5_GNP hydrogels demonstrated rounded shapes and was well-distributed in the hydrogels. GE_GNP has the highest cell viability (92.5 ± 3.54%) compared to GPVA3_GNP and GPVA5_GNP (91.5 ± 2.12% and 90.5 ± 0.71%), respectively. The pre-mixed HDFs with the hydrogels were revealed to have the highest cell viability in GE_GNP (96 ± 1%) with no significant difference between GPVA3_GNP and GPVA5_GNP (95.3 ± 1.53% and 95 ± 2%). However, the cell viability results indicated no significant difference between cell seeding and pre-mixed approaches. Moreover, the scanning electron microscopy (SEM) observation for cell interaction on the scaffold and pre-mix in the scaffold are evaluated in Figure 7b. The morphology of HDFs on the gelatin–PVA hydrogels showed as flat without forming spindle, and an absence of filopodia. No morphology changes were detected for HDFs on the surface of GE_GNP, GPVA3_GNP, and GPVA5_GNP. However, in the pre-mixed gelatin–PVA hydrogels, the presence of attached HDFs between the structural pores of the hydrogels was demonstrated.

### 3.7. 3D-Bioprinting Assessment

**Figure 8:**
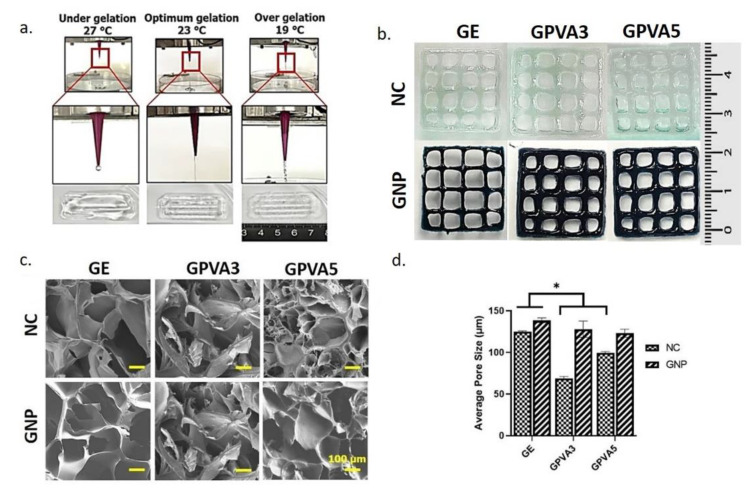
Assessment of the potential bioinks using the 3D-bioprinting fabrication technique: (**a**) printability assessment, (**b**) gross appearance of the 3D-printed hydrogels, (**c**) SEM images of the 3D-printed hydrogels, and (**d**) average pore sizes of the 3D-printed hydrogels. Whereas, * indicates the significant difference.

The 3D-bioprinting assessment of the formulated hydrogels as potential bioinks for future use via 3D-bioprinting under several different temperatures (Figure 8a) indicated 23 ± 2 °C as the optimum printing temperature. However, at temperatures of 27 ± 2 °C and 19 ± 2 °C, the gels are under gelation and over gelation states, which are not suitable for printing. Figure 8b shows the gross appearance of the 3D-printed hydrogels for GE_NC, GPVA3_NC, GPVA5_NC, GE_GNP, GPVA3_GNP, and GPVA5_GNP. The SEM micrographs of GPVA hydrogels, non-crosslinked and crosslinked, revealed that they have heterogenic porous structure, via the cross-sectional SEM images in Figure 8c. The average pore sizes of the SEM images were calculated using ImageJ Software. According to Figure 8d, the 3D-printed hydrogels have acceptable average pore sizes, which are slightly higher than the pore sizes of the injectable hydrogels in Figure 6g. Moreover, crosslinked hydrogels have higher average pore size compared to the non-crosslinked hydrogels. GE_GNP was revealed to have the highest average pore size, which is 136.68 ± 63.31 μm, followed by GPVA3_GNP, GPVA5_GNP, GE_NC, GPVA3_NC, and GPVA5_NC, with average pore sizes of 121.08 ± 58.73 μm, 119.59 ± 49.34 μm, 123.69 ± 47.09 μm, 67.44 ± 22.20 μm, and 98.48 ± 28.55 μm, respectively.

## 4. Discussion

The advancements in tissue engineering were intended to overcome injured tissue that has difficulty repairing naturally [11]. Accelerating the wound healing process is critical for skin tissue engineering to avoid severe infection and the development of chronic wounds. The goal of this research is to create an injectable hydrogel with a faster polymerisation time to be used as a cellular treatment and potential bioinks candidates for future 3D-bioprinting applications. The radical concept behind this biomatrix is to use it as a one-time post-implantation cellular skin replacement. Briefly, the incorporation of cells is estimated to promote cell proliferation, thus, accelerating the wound healing process. Moreover, the hydrogels will gradually degrade inside the skin, followed by new tissue regeneration. This study successfully fabricated the hydrogels using a blending of natural and synthetic polymers, gelatin and PVA, with several formulations, as a potential cellular treatment for chronic skin injury. The addition of PVA and GNP was aimed to improve the mechanical strength of the gelatin hydrogels [43]. Crosslinked hydrogels (GE_GNP, GPVA3_GNP, and GPVA5_GNP) were found to be the optimum formulations, polymerising in three minutes at room temperature (22–24 °C). In the current investigation, a three-minute polymerisation period was selected to provide the clinician/surgeon with adequate time to place the hydrogels on the injury site prior to polymerisation [37]. Moreover, the crosslinked hydrogels become more effective to polymerise within three minutes via covalent bond of various amino-polymeric compounds of gelatin [44].

The performance of an ideal hydrogel was further assessed based on the physicochemical properties. As compared to normal skin, wounded skin usually loses a significant amount of water and moisture. Since the traditional wound dressings or artificial tissue cannot offer adequate wound drainage, hydrogel is the best candidate as a skin replacement due to its ability to hold a high capacity of fluid [45,46]. The fabricated gelatin–PVA hydrogels possess an acceptable swelling ratio, which helps to absorb the presence of excess wound exudates at the injury sites. An ideal hydrogel candidate for wound healing applications should have a water holding capacity approximately of 500%, which will prevent exudates from accumulating in the wound region and absorb water very well [47,48]. This finding was influenced by the hydrophilic properties of the gelatin and PVA polymers. However, increasing the amount of PVA in the composite scaffold results in a decrease in the ratio of hydrophilic to hydrophobic groups in the polymer blend, thereby enhancing the flexibility of the scaffold [49]. Hence, the contact angle testing of the fabricated gelatin–PVA hydrogels demonstrated that the hydrophilicity of GPVA5_GNP has the highest contact angle, as it contains the highest percentage of PVA in the hydrogel formulation. This result was also supported by the previous findings by Shitole et al., 2019, who found that the addition of gelatin to PVA resulted in an increase in contact angle, with a mean angle of 44° [50]. Furthermore, the water contact angle phenomenon is critical in polymeric matrix and surface morphology, substantially affecting the cell adhesion activity due to water solubility and hydrogen bonding [51]. Moreover, among other factors, it has been demonstrated that surface roughness influences the cell response, including cell communication and signalling. Surface roughness has a significant impact towards cell morphology, proliferation, and phenotypic expression both in vitro and in vivo [52].

Additionally, hydrogels should have a sufficient WVTR to keep the wound area at the proper moisture level. WVTR characterisation is a critical key factor for wound healing application to maintain wound moisture. The optimum level for WVTR as a potential skin substitute is below 1500 g/m^2^/h, in order to keep the wound hydrated and to avoid over-dehydration. Therefore, the fabricated gelatin–PVA hydrogels have good WVTRs, as the results are within the hydrogel range [53]. Another factor that was considered was in vitro biodegradation, as the current limitation of hydrogel is the rapid biodegradation of biomaterials post-implantation. The results of the remaining weight for the enzymatic degradation of the hydrogels are shown in Figure 3d. The crosslinked hydrogels containing PVA displayed prolonged durability compared to the gelatin-only hydrogel group. The duration for the selected hydrogel in wound healing application should be at least 14 days before entirely degrading at the implanted site [54]. The crosslinking process using a chemical crosslinker, genipin, was used to enhance the scaffolds’ stability [39]. Genipin has been established to contain antioxidant properties to expedite wound healing phases and at the same time prolong the micro stability to sustain the cell migration and differentiation from surrounded native tissues. A porous three-dimensional microstructure allows water vapor and wound exudates to pass through. The permeability of wound exudate can help to avoid the development of lesions. All of the hydrogels have irregular porosity structures with smooth pore walls. The average pore size of the hydrogels was estimated to be more than 100 μm. As demonstrated in Figure 6e,f, GPVA3_GNP and GPVA5_GNP have substantially smaller pore sizes compared to the pure gelatin hydrogel. The reduction in pore size in GPVA3 and GPVA5 hydrogels was due to the increased amount of PVA, which improved the interaction between PVA and gelatin chain molecules, thus allowing the internal structure of the hydrogel to become more compact [55].

In this study, the crosslinked gelatin–PVA hydrogel was successfully fabricated to imitate the mechanical properties of native skin and was demonstrated to be ideal for skin application. Hydrogels incorporated with PVA have higher mechanical properties than fragile gelatin, and the inclusion of PVA increases the maximum acceptable stress and strain of the bioscaffold [32]. Therefore, this study demonstrated that the compression modulus of the GPVA5_GNP hydrogel is more than 2.0 MPa. A previous study performed by Mahnama et al., 2017, proved that gelatin–PVA scaffolds that have a higher amount of PVA are able to support the maximum compression stress with a mean of 2.2 MPa [32]. Moreover, as a skin replacement candidate, the hydrogels are skin-attached products that must be able to follow the features of the skin and not tear easily when stretched. Increases in the formation of hydrogen bonds between PVA and gelatin would result in greater stiffness as well as an increased toughness and resilience in the hydrogels [56]. Based on the thermodynamic principles of elastic polymer networks, the composite supramolecular nature hydrogels resulted in a material with great resilience. As a result, Figure 4c shows that GPVA3_GNP and GPVA5_GNP have the greatest resilience compared to crosslinked gelatin hydrogels. Moreover, a finding from a previous study by Charron et al., 2019, proved that the increment of hydrogen bonds formation between PVA and gelatin would result in greater stiffness as well as increased toughness and resilience [56]. Notably, PVA has higher viscosity than gelatin. Therefore, optimisation of appropriate viscosity is crucial for future use in 3D-bioprinting to maintain cell viability post-printing. According to our study, the viscosities of the GPVA hydrogels increased according to the addition of crosslinker and PVA, as shown in Figure 4d. Moreover, it has been reported that extrusion-based bioprinters have been proven to be compatible with bioinks that have viscosities ranging from 30 to 6 × 10^7^ mPa/s [57]. 

The concentration of amine groups (mg/mL) of the hydrogels was discovered to increase with the increment of PVA concentration in non-crosslinked hydrogels. As the gels successfully crosslinked, a bluish pigmentation in the hydrogels was seen, as shown in Figure 2b, indicating the presence of amino groups in the hydrogel network, which induce the interaction between genipin and the hydrogels. However, when the concentration of amine groups decreased, it indicated that the crosslinking degree of the hydrogels was higher. As a result, the internal network structure of the scaffolds became more compact, and the pore structure became substantially smaller. The development of intra- and intermolecular crosslinking linkages occurred via the formation of a heterocyclic structure of genipin with primary amine groups of the gelatin hydrogels [34]. The crosslinking and chemical stability was also supported by TGA analysis, as shown in Figure 4d. All hydrogels showed a significant mass loss stage in the temperature range of 25–200 °C. The first mass loss stage of non-crosslinked hydrogels occurred between 25 and 140 °C, which might be related to moisture loss (about 90%) in the hydrogels. However, the first mass loss stage of crosslinked hydrogels occurred between 25 and 120 °C. The results demonstrated that the incorporation of PVA into gelatin hydrogels slightly increased their thermal stability. It could be related to the development of hydrogen bonds between PVA–gelatin chains together with the occurrence of crosslinking hydrogels. 

Based on Figure 5a, the non-crosslinked gelatin indicated the presence of vibration amide I and amide II characteristics from polypeptides, which were assigned to N-H and aliphatic C-H stretching, respectively [50,56]. However, the result demonstrated that no change was observed at the amide II peak, which confirmed the preservation of triple helix integrity after the crosslinking application. However, the result showed a weak band at 1690 cm^−1^, and an absorption peak at 1690 cm^−1^ for GPVA3_NC, GPVA5_NC, GPVA3_GNP, and GPVA5_GNP indicated the presence of C=O stretching vibration due to the present of a small amount of PVA in the hydrogel [58]. Furthermore, this result was consistent in the XRD characterisation. The crystallinity of the hydrogel samples was evaluated using XRD in Figure 5b. The patterns of the hydrogels indicated that all of the hydrogels have a similar trend of broad peaks that occur approximately in the range of 10–30° (2θ). Based on the results, the crystallinity level of GPVA3_GNP and GPVA5_GNP slightly decreased after crosslinking. We may infer that the crystallinity is mostly attributed to gelatin instead of PVA. However, there was no new peak for the composites, indicating that PVA was adequately compatible with gelatin; and these findings support the absence of a new phase within the as-synthesised polymeric matrix system [59]. This scenario indicated that the native features of selected hybrid biomaterials were successfully maintained even though they were interfered with by a natural crosslinking agent: genipin. It is essential to ensure that the targeted cells will behave accordingly with maximum performance capacities and lowest dead cells.

The evaluations of cell viability are well-known as critical phases in toxicity testing, including cell response to a toxicant or novel substance. There was no indication of cell membrane damage after treatment with PVA (3% and 5%), which clearly showed viability equivalent to the gelatin hydrogel. Based on Figure 7a,b, the cells were shown to be viable and, on GPVA3_GNP and GPVA5_GNP, with no significant toxicity. This clearly proves that the addition of PVA in the gelatin hydrogel at an optimised concentration could enhance cell adhesion and viability on the scaffold [60], as compared to our preliminary study, where the HDFs were rounded shapes after being seeded on top of the hydrogels [61]. Moreover, the HDFs on GPVA3_GNP and GPVA5_GNP presented similar morphology compared to the ones attached on the surface of GE_GNP, which indicated that there is no significant cell morphology difference between gelatin–PVA hydrogels. In addition, this formulation also offered a novel fabrication strategy as a potential bioinks using a 3D-bioprinting approach, which is necessary to enhance cell distribution in the different layers of the hydrogels. After bioprinting, the live/dead assay indicated that almost all of the HDFs were stained green and remain viable. Moreover, the morphology of the 3D-bioprinted HDFs showed no significant differences compared to the conventional pre-mixed HDFs in the hydrogels. Moreover, the live/dead cells at different layers of the hydrogels were well-illustrated using Z-stack analysis. In addition, the micropores of the hydrogels appeared to facilitate the cell adhesion, as demonstrated in Figure 7c. This result was one of the benefits we intended to accomplish when we made the hydrogel with the addition of PVA into gelatin hydrogel. 

Since our aim is to characterise the formulation of the injectable hydrogel as a potential bioinks for 3D-bioprinting, the preliminary data were obtained through an optimisation of the printing temperature and gross appearance, and an evaluation of the average pore sizes through SEM micrographs’ analysis in Figure 8. Higher concentrations of gelatin bioinks could be fabricated at room temperature due to its high viscosity and faster transition to gel-solid state. However, since this study involved the usage of 6% gelatin, the printing temperature needed to be slightly lower for the fabrication. Moreover, a previous study that used 5% of GelMA printed the bioinks at low temperature, which was 21.18 ± 0.42 °C, due to its lower solid-gel transition [62]. The gel transition is a crucial parameter for 3D-bioprinting because it will affect the scaffold quality. However, the gelatin sol-gel transition is a reversible process that would result in hydrogel melting within a few hours if cultivated in culture media under 37 °C [63]. Therefore, in this study, the gelatin was incorporated with the PVA and crosslinked with the GNP to prevent the reversible effect of the gelatin. Moreover, due to the blending of gelatin with PVA, 23 ± 2 °C is the most optimum printing temperature with a quality printed scaffold, as stated in Figure 8b. In addition, the printing temperature of the bioinks also can be controlled and maintained using the extruder temperature controller and printing bed. 

Next, the quantification of the printed hydrogels was further evaluated using SEM analysis to calculate the pore size distribution after printing. Based on Figure 8c,d, the average pore sizes decrease as the concentration of PVA increases. The pore sizes of injectable hydrogels tend to be more organised compared to the 3D-bioprinted hydrogels that have loose networks and slightly larger and interconnected pores due to a layer-by-layer bioinks deposition. The result led us to conclude that the pore sizes were significantly increased by a 3D-bioprinting fabrication technique, which can enhance the cellular activity. However, further in vitro and in vivo research are required in the near future to improve the evaluation of the efficacy of the fabricated hybrid hydrogels as functional biomaterials (injectable approach) or potential bioinks (3D-bioprinting) for chronic wound management.

## 5. Conclusions

In conclusion, an injectable gelatin–PVA crosslinked GNP hydrogel was successfully fabricated and polymerised within three minutes at room temperature (22–24 °C), resembling potential use as a cellular skin substitute (injectable hydrogel) and as a suitable candidate for bioinks (3D-bioprinting). The presence of PVA and GNP improved the mechanical properties of the gelatin hydrogel as well as improving the vitro physicochemical and biological assessment as wound dressing materials. The results revealed that biomimetic gelatin–PVA hydrogels were promising biomaterials in terms of properties such as higher hydrophilicity and water absorption capacity, which could be useful in applications such as chronic wound healing treatment due to their capability to absorb wound exudate and provide an optimal moist wound environment due to their optimum swelling properties. The hydrogels were discovered to be biocompatible towards HDFs as a cellular wound healing treatment. The live/dead assay highlighted the efficiency of the formulated hydrogels, which could potentiate cellular interaction of the implanted site. Nevertheless, further research should be conducted to validate the efficacy of the created biomatrix as wound care products capable of supporting the dynamic process of wound healing as well as for clinical translation.

## Figures and Tables

**Table 1:** Crystallinity and amorphous levels in GPVA hydrogels via XRD analysis.

Hydrogels	Crystallinity (%)	Amorphous (%)
GE_NC	41.6%	58.4%
GPVA3_NC	45.8%	54.2%
GPVA5_NC	44.7%	55.3%
GE_GNP	47.0%	53.0%
GPVA3_GNP	39.5%	60.5%
GPVA5_GNP	43.6%	56.4%

**Table 2:** Elemental analysis with EDX. All hydrogels possessed different elemental compositions including oxygen, carbon, and nitrogen.

Sample	C (%)	O (%)	N (%)
GE_NC	60.9 ± 0.30	18.5 ± 0.21	20.6 ± 0.33
GE_GNP	60.1 ± 0.30	22.5 ± 0.20	17.4 ± 0.31
GPVA3_NC	66.9 ± 0.60	14.9 ± 0.18	18.2 ± 0.58
GPVA3_GNP	60.3 ± 3.48	19.0 ± 2.20	20.7 ± 4.11
GPVA5_NC	68.2 ± 0.69	18.8 ± 0.43	21.0 ± 0.72
GPVA5_GNP	58.7 ± 7.53	21.2 ± 5.92	20 ± 9.17

## Data Availability

Not applicable.
